# Polyphenol-rich strawberry extract (PRSE) shows *in vitro* and *in vivo* biological activity against invasive breast cancer cells

**DOI:** 10.1038/srep30917

**Published:** 2016-08-08

**Authors:** Stefano Amatori, Luca Mazzoni, Josè Miguel Alvarez-Suarez, Francesca Giampieri, Massimiliano Gasparrini, Tamara Yuliett Forbes-Hernandez, Sadia Afrin, Alfredo Errico Provenzano, Giuseppe Persico, Bruno Mezzetti, Augusto Amici, Mirco Fanelli, Maurizio Battino

**Affiliations:** 1 Molecular Pathology Lab. “PaoLa”, Dept. of Biomolecular Sciences, University of Urbino “Carlo Bo”, Fano (PU), Italy; 2 Fondazione Umberto Veronesi, Milano, Italy; 3 Dipartimento di Scienze Cliniche Specialistiche ed Odontostomatologiche, Sez. Biochimica, Università Politecnica delle Marche, Ancona, Italy; 4 Dipartimento di Scienze Agrarie, Alimentari e Ambientali, Università Politecnica delle Marche, Ancona, Italy; 5 Escuela de Medicina Veterinaria y Zootecnia, Facultad de Ciencias de la Salud, Universidad de Las Américas (UDLA), Quito, Ecuador; 6 Area de Nutrición y Salud, Universidad Internacional Iberoamericana (UNINI), Campeche, C.P.24040, Mexico; 7 Scuola di Bioscienze e Medicina Veterinaria, Università di Camerino, Camerino, Italy; 8 Centre for Nutrition & Health, Universidad Europea del Atlantico (UEA), Santander, Spain

## Abstract

We describe the biological effects of a polyphenol-rich strawberry extract (PRSE), obtained from the “Alba” variety, on the highly aggressive and invasive basal-like breast cancer cell line A17. Dose-response and time-course experiments showed that PRSE is able to decrease the cellular viability of A17 cells in a time- and dose-dependent manner. PRSE effect on cell survival was investigated in other tumor and normal cell lines of both mouse and human origin, demonstrating that PRSE is more active against breast cancer cells. Cytofluorimetric analysis of A17 cells demonstrated that sub-lethal doses of PRSE reduce the number of cells in S phase, inducing the accumulation of cells in G1 phase of cell cycle. In addition, the migration of A17 cells was studied monitoring the ability of PRSE to inhibit cellular mobility. Gene expression analysis revealed the modulation of 12 genes playing different roles in the cellular migration, adhesion and invasion processes. Finally, *in vivo* experiments showed the growth inhibition of A17 cells orthotopically transplanted into FVB syngeneic mice fed with PRSE. Overall, we demonstrated that PRSE exerts important biological activities against a highly invasive breast cancer cell line both *in vitro* and *in vivo* suggesting the strawberry extracts as preventive/curative food strategy.

In the last decades, the ability of phytochemicals to modulate apoptosis signaling pathways has attracted increasing attention as an anti-cancer agent^1^. The richest dietary sources of these bioactive compounds are fruits and vegetables, and their intake has been correlated with a decreased risk of developing several chronic pathologies, including cardiovascular and neurodegenerative diseases^2^,^3^, obesity^4^, diabetes^5^, infections^6^, skin diseases^7^ and cancer^8^, including breast cancer^9^.

Among fruits, there is growing interest in berries, and in particular strawberries (*Fragaria* x *ananassa* Duch.), due to their nutritional quality and to the numerous bioactive compounds they contain^10^. Compared with other non-berry fruits, the strawberry is a rich source of folate^11^, vitamin C and several phytochemicals that can influence the nutritional and organoleptic qualities of this fruit^12^,^13^. Its healthy effects are attributed to high levels of antioxidant compounds, most of which are phenolic compounds such as anthocyanins, flavonols, flavanols, condensed tannins (proanthocyanidins, ellagitannins, and gallotannins), hydroxybenzoic and hydroxycinnamic acid derivatives, and hydrolyzable tannins^13^,^14^. The role of strawberry bioactive compounds on cancer prevention seems to involve different mechanisms, which have not yet been fully elucidated; therefore, further investigations are needed to clarify the roles of the different strawberries phytochemicals against cancer cells. Several studies on extracts of strawberries, raspberries, and other fruits and berries, did not found any correlations between the content of some phytochemicals and inhibition of cancer cell proliferation^15^,^16^. Different studies indeed have shown that the complex mixtures of phytochemicals present in fruits and vegetables are more effective than their individual constituents in preventing cancer, through both additive and synergistic effects^17^,^18^. For this reason, it is important to study potential anticancer activity of fruits and vegetables using whole extracts containing all phytochemicals, not only using purified molecules or fractions enriched in certain classes of molecules.

The antioxidant capacity has been considered for years as a first line defense against the earlier stages of the mutagenesis process, through the capacity of these compounds to scavenge ROS species decreasing DNA oxidative damage, to stimulate antioxidant enzymes and to enhance DNA repairing. Several studies have recently underlined the ability of these compounds to modulate the cellular processes linked to cancer progression, such as cell proliferation, differentiation, apoptosis, cell cycle arrest, intracellular communication, inflammation and angiogenesis. Nevertheless, only few studies exist on the anticancer effect of strawberries and, in particular, against breast cancer^19^. Breast cancer represents the most common neoplastic disease among women worldwide, with about 1.67 million new cancer cases in 2012 (25% of all cancers), and is the second leading cause of cancer death among women in developed regions (198,000 dead, 15.4%)^20^.

Evidence supports the idea that tumor growth, recurrence and metastasis formation are dependent on cells with self-renewal properties, termed cancer initiating cells (CICs) or cancer stem cells (CSCs)^21^. A17 cells are a highly tumorigenic and invasive cell line, established from an FVB/neuT transgenic mammary tumor, displaying properties of CICs and basal-like breast cancer^22^,^23^,^24^,^25^,^26^. It has been demonstrated that A17 cells exhibit a stemness-related gene signature virtually identical to that of mesenchymal stem cells and are able to induce secondary tumor lesions due to metastasis formation^25^. In this study we investigated the biological activity of “Alba” strawberry extract on breast cancer, with a particular focus on the A17 cellular model.

## Results

### Phytochemical analysis of PRSE

The nutritional and phytochemical composition of PRSE was characterized by analyzing vitamin C content, phytochemical levels and the total antioxidant capacity (TAC) of the extract. Results are reported in Table 1 and show, according to our previous studies^27^, that “Alba” extract presents high levels of Vitamin C (0.57 mg/g) and phytochemical compounds measured as Total Phenolic Content (TPH) (2.26 mg GAEq/g), Total Flavonoids Content (TFC) (0.61 mg CEq/g) and Total Anthocyanin Content (ACY) (0.46 mg Pg-glcEq/g).

However, TFC and ACY values are similar to those of other strawberry cultivars, while the level of total phenolic compounds results very high if compared to commercial strawberry varieties previously analyzed^27^,^28^.

Moreover, the total antioxidant capacity of PRSE was quantified showing relevant values by both Trolox Equivalent Antioxidant Capacity (TEAC) (17.97 μmol TE/g) and Ferric Reducing Antioxidant Power (FRAP) (12.62 μmol TE/g) assays (Table 1).

### Biological effects of PRSE on A17 cells

**Figure 1:**
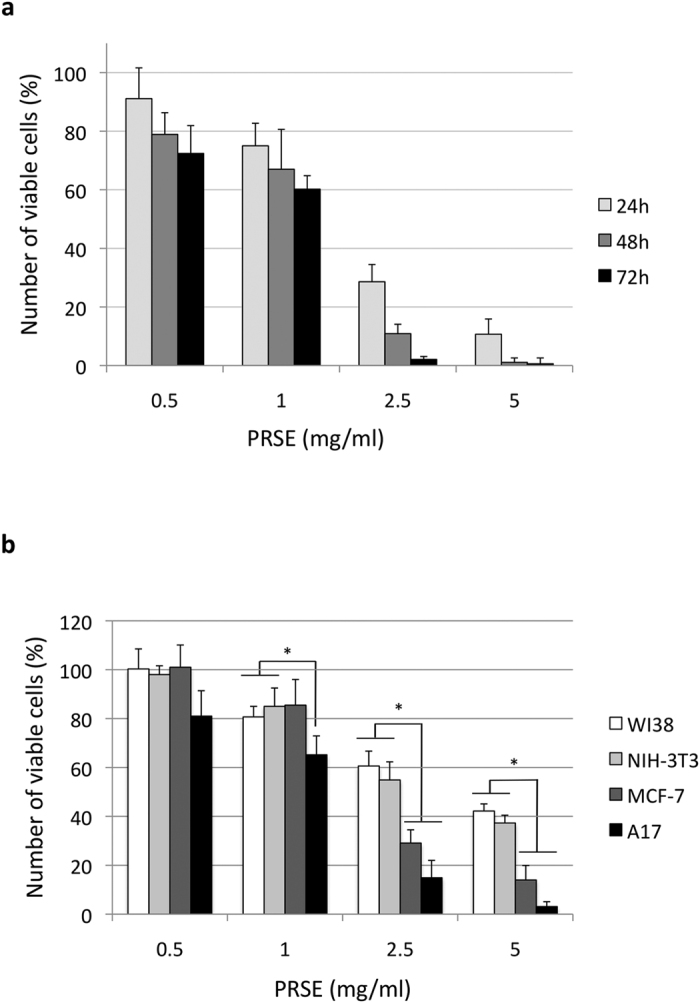
Effects induced by PRSE on cellular viability. (**a**) Dose-response and time-course experiments on A17 cells. Cells were treated with the reported concentrations of PRSE for 24 h, 48 h or 72 h. After treatment cell viability was evaluated by Trypan blue dye exclusion assay and calculated as percentage compared to the untreated control. (**b**) Dose-response experiments on normal (WI38 and NIH-3T3) and breast cancer (MCF-7 and A17) cell lines. Cells were treated with the reported concentrations of PRSE for 48 h. After treatment cell viability was evaluated by Trypan blue dye exclusion assay and calculated as percentage compared to the untreated control. Data are reported as the mean ± standard deviation of three independent experiments. *P < 0.05 respect to normal cell lines by Student’s T test.

The effects induced by the extract on the survival of A17 cells were analyzed in both dose-response and time-course experiments. Cells were treated for 24 h, 48 h or 72 h with concentrations of extract ranging from 0.5 to 5 mg/ml. Interestingly, we found that the extract reduces the survival of A17 cells in a time and dose-dependent manner (Fig. 1a).

Once characterized in A17 cells, the activity of PRSE was investigated also in normal fibroblast cell lines of both murine (NIH-3T3) and human (WI38) origin and in the human breast cancer cell line MCF-7 by dose-response studies at 48 h of treatment. Interestingly, we found that: i) A17 cells show the higher response to PRSE exposure (IC_50_ of 1.14 ± 0.29 mg/ml) and, most importantly, that ii) normal cells are significantly less sensitive to PRSE respect to cancer cell lines (mean IC_50_ of 3.24 ± 0.14 mg/ml and 1.68 ± 0.77 mg/ml, respectively – Fig. 1b).

**Figure 2:**
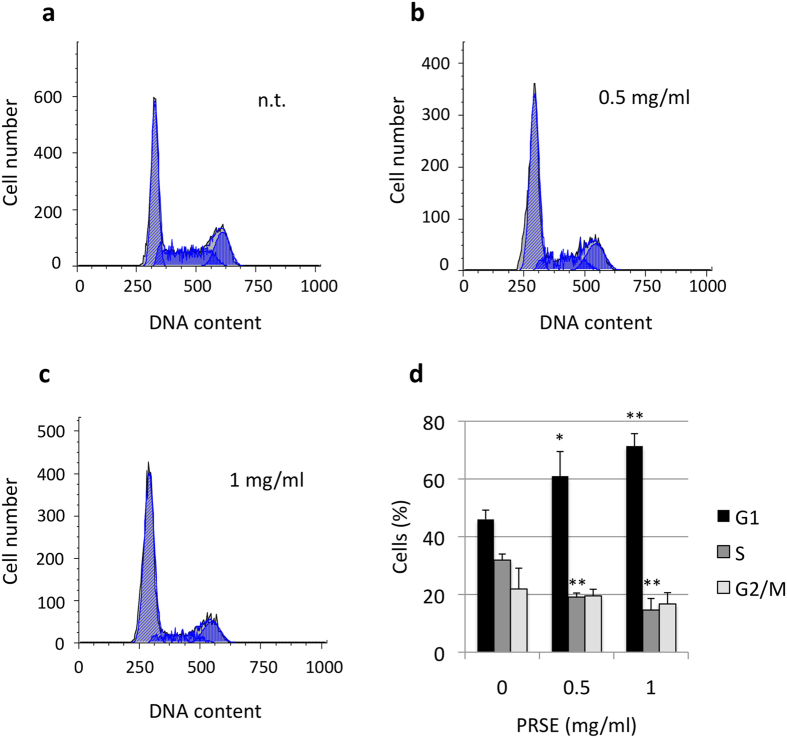
Cell cycle alterations induced by PRSE on A17 cells. A17 cells were treated for 48 h with the concentrations of PRSE reported in the figure or left untreated. Cells were stained with propidium iodide and the DNA content of cells was analyzed by a cytofluorimeter. (**a–c**) Representative examples of cell cycle distribution. (**d**) Cell cycle percentages calculated by FlowJo 8.6.3 software. Data are reported as the mean ± standard deviation of three independent experiments. *P < 0.05, **P < 0.01 respect to not treated cells by Student’s T test.

**Figure 3:**
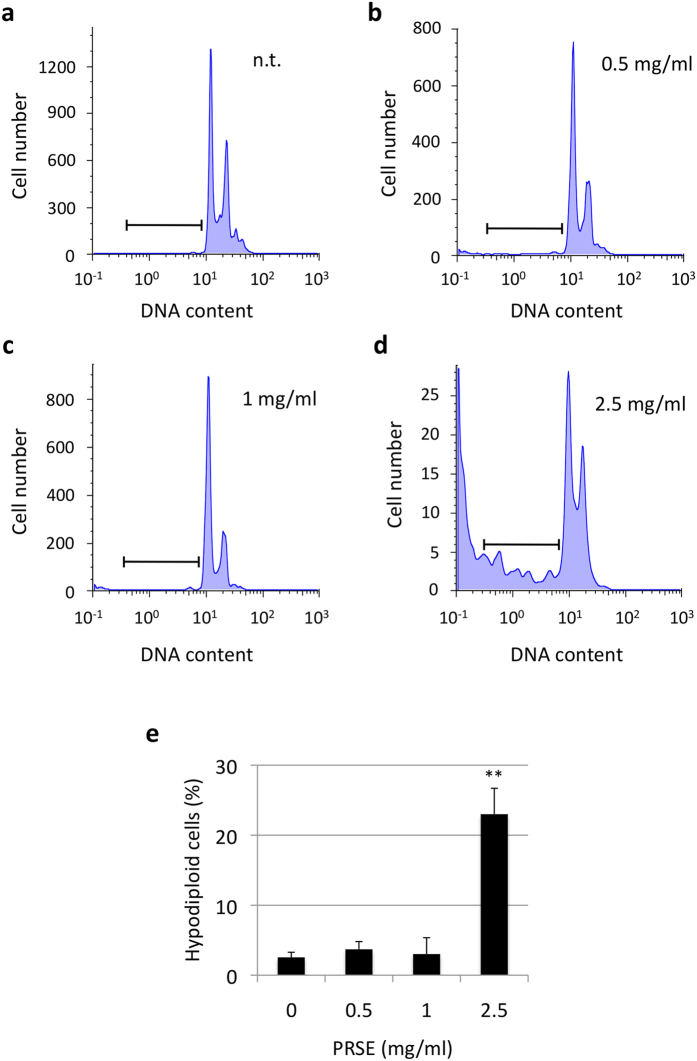
Percentage of hypodiploid cells in PRSE-treated A17 cells. A17 cells were treated for 48 h with the concentrations of PRSE reported in the figure or left untreated. Cells were stained with propidium iodide and the DNA content of cells was cytofluorimetrically analyzed. Experiments were performed in triplicate. (**a–d**) Representative examples of untreated and treated cells. (**e**) Percentages of hypodiploid cells calculated by FlowJo 8.6.3 software. Data are reported as the mean ± standard deviation of three independent experiments. **P < 0.01 respect to not treated cells by Student’s T test.

Subsequently, possible alterations of cell cycle phase distribution were evaluated by cytofluorimetric analysis of propidium iodide stained cells. A17 cells treated for 48 h with different concentrations of PRSE showed, at the concentrations of 0.5 and 1 mg/ml of PRSE treatments, a reduction of cells in S phase and a concomitant increase of cells in G1 phase of the cell cycle (Fig. 2). Moreover, treatments with higher doses of PRSE (2.5 mg/ml) drastically change the biological response of A17 cells, hindering the cell cycle analysis but showing the appearance of a hypodiploid cellular subpopulation (Fig. 3).

**Figure 4:**
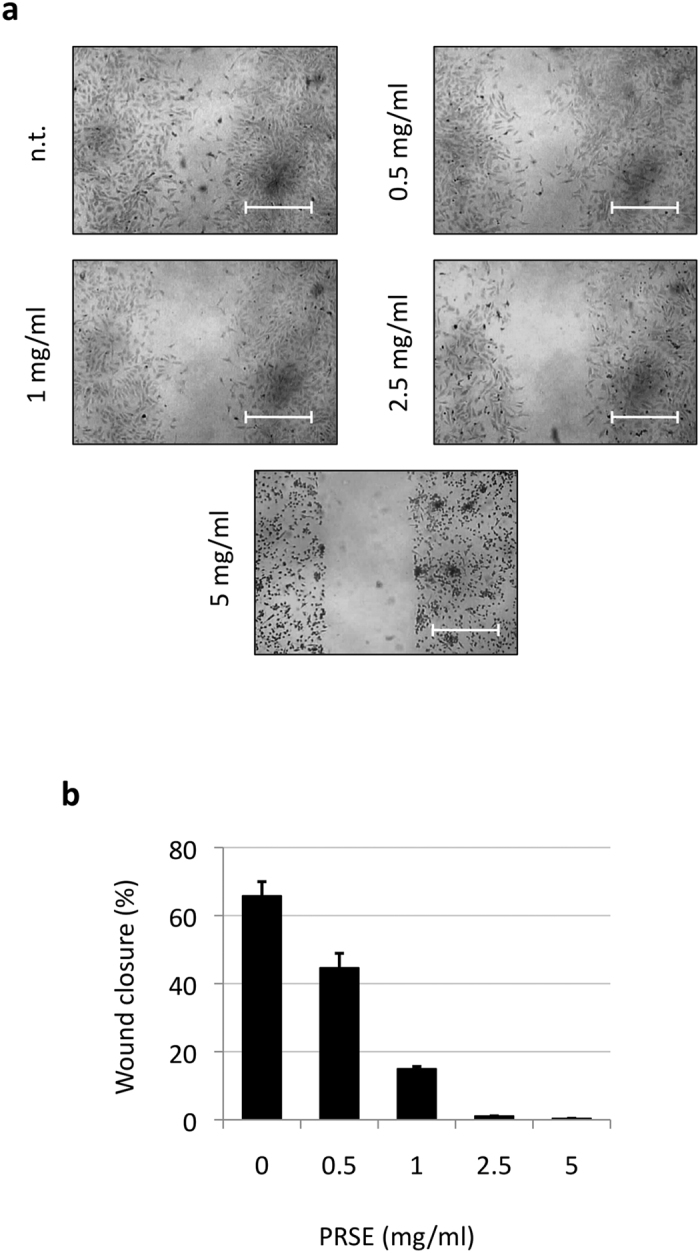
PRSE effect on the migration ability of A17 cells. (**a**) Wound-healing assay of A17 cells untreated or treated with different concentrations of PRSE. All samples were analyzed after 48 h of treatments. Representative images from three independent experiments are shown. (**b**) Quantification of the percentage of wound closure by Image J software. Data are reported as the mean ± standard deviation of three independent experiments.

A17 cell line is an intriguing model of study because of its highly aggressive and invasive phenotype^25^. In consideration of the critical role of cellular migration on the multistep process of tissue invasion^29^ we decided to investigate the possible interference of PRSE on the mobility of A17 cells by wounding assay. To avoid cell turnover that could mimic the cell migration, cells were starved during this assay. After 48 h of culture, A17 cells not subjected to treatments were able to migrate and partially fill the wound empty area while exposure to PRSE inhibited the cell migration in a dose-dependent manner (Fig. 4a). Notably, the inhibition of wound closure was monitored starting from the lowest concentration of PRSE tested (0.5 mg/ml), and appears almost completely inhibited starting from a concentration of 2.5 mg/ml (Fig. 4a). A quantitative analysis of wound closure was carried out using the NIH Image J software (Fig. 4b).

### PRSE modulation of gene expression

**Figure 5:**
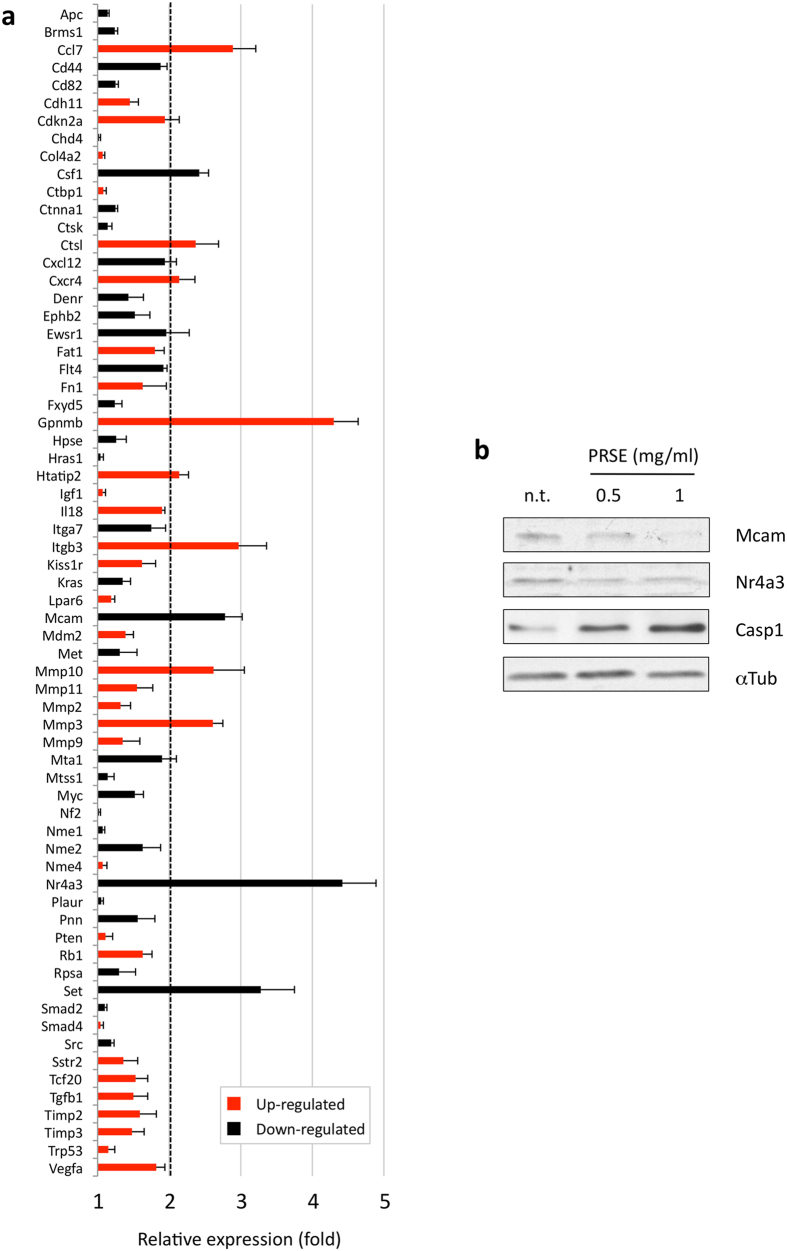
Effects of PRSE on the expression of genes involved in migration, adhesion and invasion processes in A17 cells. (**a**) Screening by PCR array of 84 mouse genes involved in the migration, adhesion and invasion processes (only the 66 genes amplified are displayed). A17 cells were treated for 48 h with sublethal doses of PRSE or left untreated. After RNA extraction and retrotranscription, the resulting cDNA was applied in the array and amplified. Data are reported as fold compared to control untreated A17 cells. Genes showing a regulation exceeding the 2-fold criteria were considered as differentially expressed. Data are reported as the mean ± standard deviation of three independent experiments. The transcripts of the following genes were not amplified: Cdh1, Cdh6, Cdh8, Cxcr2, Elane, Etva, Ewsr1, Fgfr4, Hgf, Il1b, Kiss1, Mmp13, Mmp7, Mycl, Rorb, Timp4, Tnfsf10 and Tshr. (**b**) Western blot analysis of total cell lysates obtained from PRSE-treated and untreated (n.t.) A17 cells.

The biological effects mediated by the extract on A17 cells were also investigated at the molecular level by analyzing the expression of a panel of genes known to be involved, with different roles, in the cellular migration, adhesion and invasion processes. For this purpose, the level of 84 different mouse gene transcripts was analyzed by applying a quantitative RT-PCR array on A17 cells untreated or treated for 48 h with sub-lethal doses of PRSE. Among the 84 gene transcripts screened, 12 genes showed a regulation exceeding the 2-fold criteria compared to the untreated control (Fig. 5a). Genes down-regulated include the colony stimulating factor 1 (Csf1, −2.42 fold), the melanoma cell adhesion molecule (Mcam, −2.78 fold), the nuclear receptor subfamily 4, group A, member 3 (Nr4a3, −4.42 fold) and SET nuclear proto-oncogene (SET, −3.38 fold). The transcripts showing the strongest up-regulation were glycoprotein nmb (Gpnmb, 4.30 fold), Integrin beta 3 (Itgb3, 2.97 fold) and the chemokine (C-C motif) ligand 7 (CCl7, 2.89 fold). In addition, also cathepsin L (Ctsl, 2.37 fold), chemokine (C-X-C motif) Receptor 4 (Cxcr4, 2.14 fold), HIV-1 tat interactive protein 2 (Htatip2, 2.14 fold), and matrix metalloproteinases 10 and 3 (Mmp10 and Mmp3, 2.62 and 2.61 fold, respectively) showed an up-regulation exceeding the 2-fold criteria. Notably, 18 out of 84 transcripts present in the PCR array were not amplified (see legend of Fig. 5a).

Subsequently, two genes showing significant modulation, Mcam and Nr4a3, were further investigated at protein level by western blotting (Fig. 5b). Densitometric analysis of the bands of the two proteins was performed and normalized to alpha-tubulin expression, confirming the downregulation of Mcam (−1.89 fold at 0.5 mg/ml and −4.25 fold at 1 mg/ml of extract) and, at a less extent, of Nr4a3 (−2.37 fold at 0.5 mg/ml and −1.54 fold at 1 mg/ml of extract). In addition, caspase-1 levels were also analyzed by western blotting showing the upregulation of the protein at both 0.5 mg/ml (+1.89 fold) and 1 mg/ml (+4.16 fold) of PRSE (Fig. 5b).

### *In vivo* evaluation of PRSE activity

**Figure 6:**
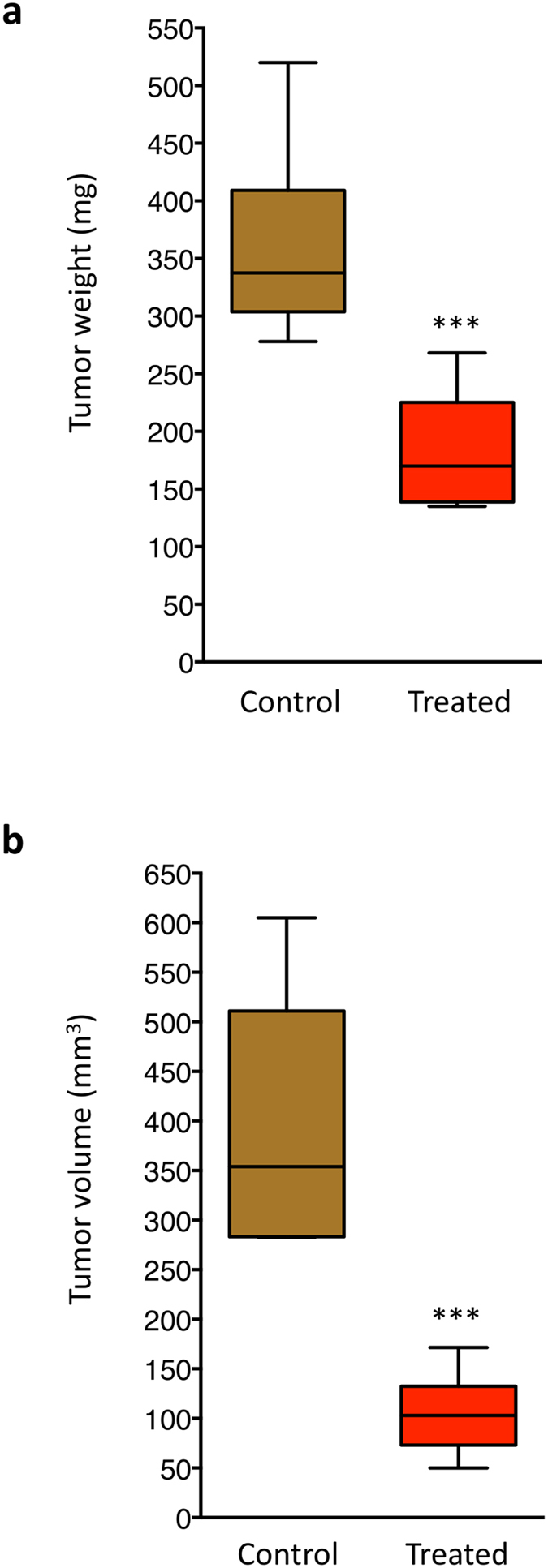
Effect of PRSE on A17 tumors in mice. Ten mice (fed with 15% strawberry extract-enriched food) were housed one mouse/cage, while ten mice (fed with regular food) were housed in two cages with free access to water and food. Upon the 8^th^ week of age, all the mice underwent tumor challenge with 2 × 10^5^ A17 cells. After cells injection mice were orally treated for further 5 weeks, at the end of which tumors were withdrawn and both tumor weight (**a**) and tumor volume (**b**) were measured. ***P < 0.001 by Student’s T test.

A17 cells were used to generate an orthotopic model of breast cancer in syngeneic female FVB/N mice. Four weeks mice were fed with 15% strawberry extract-enriched food or regular food as control (ten mice for each group). Upon the 8^th^ week of age, all the mice underwent tumor challenge while animals of each group continued to receive their diets. After 5 weeks, tumors were withdrawn, measured using a caliper and weighted. Results show a significant reduction in both tumor weight (Fig. 6a) and tumor volume (Fig. 6b) in mice fed with PRSE.

## Discussion

The strawberry (*Fragaria* x *ananassa*) was chosen as the model in this study for its high content of antioxidants and bioactive compounds, as well as for its high availability for food industry and fresh consumption^13^. In the last decades, growing interest has been focused on the antioxidant capacity of strawberries so that TAC is considered a quality parameter and an indicator of bioactive compounds present in the fruit. The antioxidant capacity of the strawberry indicates that its consumption could contribute to prevent and reduce oxidative reactions that cause negative effects on human health, playing different roles in the development of chronic diseases and cancers.

Moreover, even if the high TAC of strawberries has been proved, it has also been demonstrated that this parameter is strongly influenced by the strawberry genetic background and is strictly related to the presence of oxygen radical scavengers such as Vitamin C and polyphenols^11^,^27^,^28^,^30^,^31^. Among polyphenols, anthocyanins are quantitatively the most important phenolic compounds in strawberries^13^, so that more than 25 different anthocyanin pigments have been described from different varieties and selections^32^. Analyses of TPH, ACY and TFC in the “Alba” cultivar in the present study confirm the high content of phytochemicals of this strawberry.

Strawberries are important also for their high content of Vitamin C, which is even higher than that of citrus fruit. Vitamin C content is an essential parameter due to the high number of biological roles that it plays in humans, lowering the incidence of cardio-and cerebro-vascular diseases^33^, of several cancers^34^, and other health disorders such as lead toxicity^35^. When evaluating the Vitamin C content in strawberries, it is important to consider that the molecule is very labile and in adverse conditions undergoes oxidation, depending on several factors such as temperature, water and pH^36^.

The choice of using “Alba” strawberries in this study was also based on the very interesting results obtained in recent years using this cultivar, both *in vitro*^37^,^38^ and *in vivo*^39^,^40^.

As shown above, the “Alba” strawberry extract is able to strongly decrease cellular viability of the highly tumorigenic stromal cell line A17, which is characterized by mesenchymal features and metastasis formation ability^26^,^41^.

In particular, it is possible to distinguish two different biological behaviors: i) a cytostatic-like effect at low doses of PRSE and ii) an acute toxic effect at higher doses. The capacity of the strawberry extract to activate the apoptotic process, as well as its anti-cancer potential in other cancer models, was already characterized by other authors^19^. However, although these observations are in accordance with the increased hypodiploidy observed in this study at high PRSE doses, the analysis of DNA fragmentation by laddering assay did not show the induction of the apoptotic internucleosomic cleavage of DNA (data not shown). Interestingly, we observed an increased expression level of the precursor form of caspase-1 that, together with the monitored hypodiploidy, could suggest an activation of the apoptotic pathway at a very early stage, and thus below the detection limit of the experimental approaches used. In addition, we found no evidence of caspase-3 modulation and/or activation (data not shown).

We hypothesized that sub-lethal doses of strawberry extract could be able to inhibit the known invasive potential of A17 breast cancer cells. Thus, the effect of PRSE on the cellular migration, which is known to play a pivotal role in invasion, was first analyzed at a biological level by wounding healing assay. The observation that low doses of “Alba” PRSE were sufficient to block cell migration gave us the rationale to extend the study at the molecular level. The subsequent characterization of the expression levels of genes involved in the cellular migration, adhesion and invasion processes allowed us to obtain an overall view of changes that take place in several pathways. In particular, the down-regulation of a subgroup of genes (Csf1, Mcam, Nr4a3 and Set) supports the anti-invasion effect of the PRSE extract because high expression levels of these genes are often associated to the invasive phenotype of cells. In the context of human breast cancer it has been already reported that: i) high expression of Csf1 is correlated to metastasis formation and thus has been proposed as negative prognostic factor; ii) up-regulation of human Mcam (named Muc18 in humans) promotes motility, invasiveness and tumorigenesis of human breast cancer cells; iii) high expression levels of Nr4a3 were found correlated with increased risk of developing distant metastasis in triple-negative breast cancer patients; iv) Set nuclear proto-oncogene (named also I2pp2a) is one of the genes down-regulated by the mushroom *Ganoderma lucidum* extract and is involved in the suppression of breast-to-lung cancer metastasis^42^,^43^,^44^,^45^. Interestingly, the modulation of Mcam and Nr4a3 was confirmed also at the protein level, further suggesting a role of these proteins in the effect induced by PRSE on A17 cells.

Likewise, we found two genes whose monitored up-regulation, as a consequence of PRSE treatment, can be consistent with an anti-invasion effect: i) Htatip2 (named also Tip30), a putative metastasis suppressor gene which is inversely correlated with lymph node metastasis in breast cancer patients, and ii) Gpnmb (also named Osteoactivin/HGFIN), a gene with a controversial role in the metastatic process^46^,^47^.

Otherwise, it was not possible to find any correlation between the known activity of the remaining six genes found up-regulated by PRSE (Ccl7, Ctsl, Cxcr4, Itgb3, Mmp19, Mmp3) since their functions are generally associated to the acquisition of invasive features^44^,^48^,^49^,^50^,^51^.

As already stated, the role of strawberry bioactive compounds on cancer prevention seems to involve different mechanisms of action that are still unclear. Previous studies indeed indicate that the biological activity exerted by berries against cancer cells is probably mediated by the synergy between different compounds suggesting the involvement of several molecular pathways. To date, only few studies have tried to investigate the molecular basis of strawberry activity against breast cancer cells. For example, the role of p73 in triggering apoptosis of p53-null cells has been recently suggested^19^. Our study indicates the involvement of other genes known to play key roles in cellular invasion, adhesion and migration.

Notably, cell survival studies show that the effect of the extract is significantly higher in breast cancer cell lines, and in particular A17 cells, with respect to normal cells, suggesting a therapeutic window for PRSE *in vivo*. This observation is in accordance with previous studies on mice that showed efficacy of strawberry methanolic extract against Ehrlich ascites carcinoma (EAC)^19^. We thus explored the efficacy of PRSE in inhibiting tumor formation *in vivo* exploiting the intriguing orthotopic breast cancer model generated by injection of A17 cells directly into the mammary gland of mice, finding a strong reduction of both tumor size and tumor volume in mice fed with PRSE from 4 weeks before tumor challenge.

Although the mechanism by which the strawberry extract exerts its biological effect is not completely understood at the molecular level, as well as which component plays the major antineoplastic role, our results suggest an interesting anti-invasive potential of “Alba” PRSE against breast cancer cells both *in vitro* and *in vivo*. Further studies will be necessary to unravel the pathways involved in the biological effects of PRSE and to elucidate the molecular basis of strawberry extract action, as well as to shed light on the possible introduction in the diet of “Alba” and, more in general, strawberries as a useful nutrient to limit (or prevent) tumor formation.

## Methods

### Preparation of PRSE

Strawberry fruits of the “Alba” variety were collected in the experimental fields of the Agricultural Faculty of “Università Politecnica delle Marche” located in Agugliano (AN), in central east Italy (43°31′60″ N-13°22′60″ E). Fruit samples from the selected variety were hand-picked at the same day-time on different days, corresponding to the ripening times of the selected clone, from the second to the fourth picking. Fruit samples were selected for homogenous fruit, avoiding unripe, wounded or shriveled fruits. Within 2 h after harvest, whole fruits were stored at −20 °C before analyses. For the evaluation of total antioxidant capacity (TAC), total phenolic content (TPH), total anthocyanin content (ACY) and total flavonoid content (TFC), a methanolic extract was prepared via homogenization. Frozen strawberries were thawed for 60 min at 4 °C. Ten gram aliquots of the fruits were added to 100 mL of the extraction solution, consisting of methanol/milliQ water/concentrated formic acid (80:20:0.1 v/v), and fruits were homogenized using an Ultraturrax T25 homogeniser (Janke & Kunkel, IKA Labortechnik, Staufen, Germany) for 2 min. Extraction was maximized by stirring the suspension for 2 h in the dark at room temperature (RT), then the tubes were centrifuged at 3500 rpm for 15 min, in two sequential times, to sediment solids. Supernatants were filtered through a 0.45 μm Minisart filter (PBI International, Milan, Italy), transferred to 5.0 ml amber glass vials and stored at –20 °C until analysis. Methanolic extract was concentrated through a rotary evaporator and stored in aliquots at −80 °C for subsequent experimental procedures. Vitamin C analysis was conducted as described^28^.

### PRSE analysis

Two methods were used for the determination of the antioxidant capacity (TAC) of strawberry extracts: the Trolox Equivalent Antioxidant Capacity (TEAC) and the Ferric Reducing Antioxidant Power (FRAP). TEAC assay consists in the quantification of strawberry extract free radical scavenging activity against 2,2-azinobis-(3-ethylbenz-thiazoline-6-sulfonate) radical cation (ABTS^+^), using Trolox (6-Hydroxy-2,5,7,8-tetramethylchroman-2-carboxylic acid) as reference standard^52^,^53^. Briefly, 1 ml of the ABTS^+^ radical solution was mixed with 10 μl of reagent (strawberry extract or standard). The analysis solution was vortexed for 20 seconds and, after 1–3 min, spectrophotometrically analyzed at 734 nm measuring the color inhibition of the ABTS^+^ radical. FRAP assay was carried out according to the protocol proposed by Deighton and coworkers^54^, with slight modifications^55^.

Total phenolic content (TPH) of the strawberry extracts was determined using the Folin-Ciocalteu colorimetric method, as modified by Slinkard and Singleton^56^. Briefly, 100 μL of sample (milliQ water, water diluted strawberry extracts or gallic acid standard solutions) were added to 500 μL of Folin-Ciocalteau reagent previously diluted in water (dil. 1/10) and kept at 4 °C, in the dark. The mixture was incubated for 1 to 8 min at RT, then 400 μL of 0.7 M sodium carbonate were added and the mixture was vortexed. The solution was incubated for 2 h at RT, in the dark and spectrophotometrically analyzed at 760 nm.

The total anthocyanin (ACY) content of the strawberry extracts was determined using a modified pH differential method^57^, while total flavonoid content (TFC) was determined by using a colorimetric method^58^,^59^.

Vitamin C content was measured through HPLC analysis immediately after the extraction procedure. The HPLC system comprised a Jasco (Jasco Inc., Easton, USA) PU-2089 Plus controller and a Jasco UV-2070 Plus ultraviolet (UV) detector set at absorbance of 260 nm. An isocratic elution with 50 mM potassium phosphate at pH 3.2 was performed by means of a Supelcosil LC8 150 × 4.6 mm HPLC column (Sigma-Aldrich S.r.l. Milan, Italy)^60^. Quantification of the vitamin C was carried out through a comparison with pure vitamin C calibration curve. All the analyses were conducted in triplicate.

### Cell culture

The stromal cell line A17 was isolated from a murine model of mammary carcinoma induced by the overexpression of HER-2/neu transgene in the epithelial compartment of mammary glands in FVB mice, line 233^25^. A17 cells were cultured in high-glucose Dulbecco’s Modified Eagle Medium (DMEM - Euroclone, Pero, Italy), supplemented with 20% fetal bovine serum (Gibco - Hyclone, South Logan, UT, USA), 1% penicillin-streptomycin and 1% glutamine.

WI38, NIH-3T3 and MCF-7 cell lines were kindly provided by Prof. Pier Giuseppe Pelicci (European Institute of Oncology - Milan, Italy, 2016) and cultured in high-glucose Dulbecco’s Modified Eagle Medium (DMEM - Euroclone, Pero, Italy), supplemented with 10% fetal bovine serum (Gibco - Hyclone, South Logan, UT, USA), 1% penicillin-streptomycin and 1% glutamine.

All the cell lines were grown in a humidified atmosphere at 37 °C and 5% CO_2_ as previously described^61^,^62^.

### Cell viability

Dried PRSE, stored in aliquots at −80 °C, was diluted in culture medium immediately before use. Cells were seeded in triplicate in 6-well plates 16 hours before PRSE addition. After treatment with different concentrations of extract, cell viability was measured by a TC10 automatic cell counter (BioRad, Hercules, CA, USA) and compared with an untreated control to estimate the cell viability. The 50% inhibitory concentration (IC_50_) value was calculated with CompuSyn software (ComboSyn, Inc., Paramus, NJ, USA)^63^. Data are reported as mean (±SD) resulting from three independent experiments.

### Cell cycle analysis

Cell cycle was analyzed using the propidium iodide staining procedure as previously reported^64^. Cells were fixed in ice-cold 70% ethanol and stained using a propidium iodide staining solution (50 mg/ml). Cytofluorimetric acquisitions were carried out with a PAS flow cytometer (Partec, Münster, Germany) and sample analysis conducted using FlowJo 8.6.3 software (Tree Star, Inc., Ashland, OR, USA). Cell cycle percentage values were calculated using a Watson pragmatic model.

### Wounding assay

Wounding assay was performed as previously described on the same cellular model (A17)^65^. Briefly, a linear wound was produced in A17 confluent cellular population by scratching the bottom of each dish with a sterile pipette tip. After wounding, cells were washed with phosphate buffer saline (PBS) and incubated in high glucose DMEM containing 0.5% FBS with different strawberry extract concentrations (0.5, 1, 2.5 and 5 mg/ml). A17 cells were allowed to migrate for additional 48 h after which images acquisition was carried out by a LeitzFluovert FU (Leica Microsystems) microscope. Remaining wound areas were determined using NIH Image J software for calculation of the percentage of wound closure. Analyses were performed in triplicate.

### Gene expression analysis and western blotting

The Tumor Metastasis RT^2^ Profiler PCR Array (Qiagen, Hilden, Germany), consisting of 84 mouse genes known to be involved in the metastasis formation process, was used to profile the biological response of A17 cells induced by the PRSE. Briefly, total RNA was extracted from treated or untreated A17 cells using RNeasy extraction kit and reverse transcribed into cDNA using an RT^2^ First Strand Kit (Qiagen). The cDNA was combined with RT^2^ SYBR Green qPCR Master Mix (Qiagen), and equally distributed (25 μl) to each well of the PCR array plate. Real-time PCR assay and subsequent data collection were performed on a Rotor-Gene 6000 robocycler (Corbett Life Science, Sydney, Australia)^66^. Transcript relative enrichments were calculated following manufacturer’s instructions (Qiagen). Experiments were performed in triplicate.

Western blotting analyses were performed as previously described^66^ using anti-CD146 (Mcam - #ab75769, lot #GR208953-3 from Abcam), anti-NOR-1 (Nr4a3 - #sc-133840, lot #G1911 from Santa Cruz Biotechnology), anti-Caspase-1 (#66441A from Pharmingen International) and anti-αTubulin (#T9026, lot #057K4842 from Sigma) antibodies. Goat anti-rabbit IgG, horseradish peroxidase conjugate (#G21234, lot #35837A) and goat anti-mouse IgG, horseradish peroxidase conjugate (#G21040, lot #83E1-1) were purchased from Molecular Probes. Elaboration of pictures and densitometric analysis were performed using ImageJ software (ImageJ 1.43u; National Institutes of Health, Bethesda, MD, USA). The data obtained by the densitometric analysis were normalized to alpha-tubulin protein levels and expressed as fold changes.

### *In vivo* evaluation of PRSE efficacy

Four weeks old female FVB/N mice were obtained from Animal Care Facilities of the University of Camerino. Ten mice (fed with 15% strawberry extract-enriched food) were housed one mouse/cage, while ten mice (fed with regular food) were housed in two cages with free access to water and food, and kept at temperature of 19–22 °C and relative humidity of 45–65% under 12 h/12 h light/dark cycle. Upon the 8^th^ week of age, all the mice were orthotopically injected with 2 × 10^5^ A17 cells. Tumor monitoring was performed twice a week by palpation. After 5 weeks, the tumors were analyzed, after resection, evaluating both weight and volume (two perpendicular diameters - *a* and *b* - on each tumor were measured using a caliper and the volumes were calculated by the V = π/6[(a + b)/2]^3^ formula). All the experimental procedures carried out in this study were in compliance with the UK Animals (Scientific Procedures) Act 1986 and associated guidelines, EU Directive 2010/63/EU, and were approved by the Ethic Committee on Animal Use of the University of Camerino (protocol number 14/2012).

## Additional Information

**How to cite this article**: Amatori, S. *et al.* Polyphenol-rich strawberry extract (PRSE) shows *in vitro* and *in vivo* biological activity against invasive breast cancer cells. *Sci. Rep.* **6**, 30917; doi: 10.1038/srep30917 (2016).

## Figures and Tables

**Table 1:** Micronutrient composition, phytochemical content and antioxidant capacity of strawberry extract.

Parameter	Quantification
Vitamin C (mg/g FW)	0.57 ± 0.03
TPH (mg GAEq/g FW)	2.26 ± 0.03
TFC (mg CEq/g FW)	0.61 ± 0.02
ACY (mg Pg-glcEq/g FW)	0.46 ± 0.01
Total antioxidant capacity:	
TEAC (μmol TE/g FW)	17.97 ± 1.13
FRAP (μmol TE/g FW)	12.62 ± 0.15

## References

[b1] Aravindaram K. & Yang N.-S. Anti-inflammatory plant natural products for cancer therapy. Planta Med. 76, 1103–1117 (2010). 20432202 10.1055/s-0030-1249859

[b2] Johnsen S. P. *et al.* Intake of fruit and vegetables and the risk of ischaemic stroke in a cohort of Danish men and women. Am. J. Clin. Nutr. 78, 57–64, 2003. 12816771 10.1093/ajcn/78.1.57

[b3] Vauzour D., Vafeiadou K., Rendeiro C., Corona G. & Spencer J. P. E. The inhibitory effects of berry-derived flavonoids against neurodegenerative processes. J. Berry Res. 1, 45–52 (2010).

[b4] Papathanasopoulos A. & Camilleri M. Dietary fiber supplements: Effects in obesity and metabolic syndrome and relationship to gastrointestinal functions. Gastroenterology 138(1), 65–72 (2010). 19931537 10.1053/j.gastro.2009.11.045 PMC2903728

[b5] Carter P., Gray L. J., Troughton J., Khunti K. & Davies M. J. Fruit and vegetable intake and incidence of type 2 diabetes mellitus: Systematic review and meta-analysis. BMJ 341, c4229 (2010). 20724400 10.1136/bmj.c4229 PMC2924474

[b6] Holt E. M. *et al.* Fruit and vegetable consumption and its relation to markers of inflammation and oxidative stress in adolescents. J. Am. Diet. Assoc. 109(3), 414–421 (2009). 19248856 10.1016/j.jada.2008.11.036 PMC2676354

[b7] Kubota M. *et al.* Anti-melanogenic compounds in Rubus croceacanthus. J. Berry Res. 4(3), 127–135 (2014).

[b8] Smith-Warner S. A. *et al.* Fruits, vegetables and lung cancer: A pooled analysis of cohort studies. Int. J. Cancer 107, 1001–1011 (2003). 14601062 10.1002/ijc.11490

[b9] El Hasasna H. *et al.* Rhus coriaria induces senescence and autophagic cell death in breast cancer cells through a mechanism involving p38 and ERK1/2 activation. Sci. Rep. 5, 13013 (2015). 26263881 10.1038/srep13013 PMC4532997

[b10] Ariza M. T. *et al.* Effects of harvest time on functional compounds and fruit antioxidant capacity in ten strawberry cultivars. J. Berry Res. 5(2), 71–80 (2015).

[b11] Tulipani S. *et al.* Folate content in different strawberry genotypes and folate status in healthy subjects after strawberry consumption. Biofactors 34, 47–55 (2008). 19706971 10.1002/biof.5520340106

[b12] Tulipani S., Mezzetti B. & Battino M. Impact of strawberries on human health: insight into marginally discussed bioactive compounds for the Mediterranean diet. Public Health Nutr. 12(9A), 1656–1662 (2009). 19689836 10.1017/S1368980009990516

[b13] Giampieri F. *et al.* The strawberry: Composition, nutritional quality, and impact on human health. Nutrition 28, 9–19 (2012). 22153122 10.1016/j.nut.2011.08.009

[b14] Giampieri F. *et al.* The potential impact of strawberry on human health. Nat. Prod. Res. 27, 448–455 (2012). 22788743 10.1080/14786419.2012.706294

[b15] Meyers K. J., Watkins C. B., Pritts M. P. & Liu R. H. Antioxidant and antiproliferative activities of strawberries. J. Agric. Food Chem. 51, 6887–6892 (2003). 14582991 10.1021/jf034506n

[b16] Olsson M. E., Gustavsson K.-E., Andersson S., Nilsson A. & Duan R.-D. Inhibition of cancer cell proliferation *in vitro* by fruit and berry extracts and correlations with antioxidant levels. J. Agric. Food Chem. 52, 7264–7271 (2004). 15563205 10.1021/jf030479p

[b17] Liu R. H. Potential synergy of phytochemicals in cancer prevention: mechanism of action. J. Nutr. 134, 3479S–3485S (2004). 15570057 10.1093/jn/134.12.3479S

[b18] Seeram N. P. *et al.* *In vitro* antiproliferative, apoptotic and antioxidant activities of punicalagin, ellagic acid and a total pomegranate tannin extract are enhanced in combination with other polyphenols as found in pomegranate juice. J. Nutr. Biochem. 16, 360–367 (2005). 15936648 10.1016/j.jnutbio.2005.01.006

[b19] Somasagara R. R. *et al.* Extracts of Strawberry Fruits Induce Intrinsic Pathway of Apoptosis in Breast Cancer Cells and Inhibits Tumor Progression in Mice. PLoS One 7(10), e470212012 (2014). 10.1371/journal.pone.0047021 PMC3468438 23071702

[b20] Ferlay J. *et al.* Cancer incidence and mortality worldwide: sources, methods and major patterns in GLOBOCAN 2012. Int. J. Cancer 136, E359–E386 (2015). 25220842 10.1002/ijc.29210

[b21] Clevers H. The cancer stem cell: premises, promises and challenges. Nat. Med. 17(3), 313–319 (2011). 21386835 10.1038/nm.2304

[b22] Montani M. *et al.* The water soluble ruthenium(II) organometallic compound [Ru(p-cymene)(bis(3,5 dimethylpyrazol-1-yl)methane)Cl]Cl suppresses triple negative breast cancer growth by inhibiting tumor infiltration of regulatory T cells. Pharmacol Res. 107, 282–290 (2016). 27038531 10.1016/j.phrs.2016.03.032

[b23] Garulli C. *et al.* Dorsomorphin reverses the mesenchymal phenotype of breast cancer initiating cells by inhibition of bone morphogenetic protein signaling. Cell Signal. 26(2), 352–362 (2014). 24280125 10.1016/j.cellsig.2013.11.022

[b24] Bisaro B. *et al.* p130Cas/Cyclooxygenase-2 axis in the control of mesenchymal plasticity of breast cancer cells. Breast Cancer Res. 14(5), R137 (2012). 23098208 10.1186/bcr3342 PMC4053116

[b25] Galiè M. *et al.* Mammary carcinoma provides highly tumourigenic and invasive reactive stromal cells. Carcinogenesis 26, 1868–1878 (2005). 15975963 10.1093/carcin/bgi158

[b26] Galiè M. *et al.* Mesenchymal stem cells share molecular signature with mesenchymal tumor cells and favor early tumor growth in syngeneic mice. Oncogene 27, 2542–2551 (2008). 17998939 10.1038/sj.onc.1210920

[b27] Tulipani S. *et al.* Ascorbate, not urate, modulates the plasma antioxidant capacity after strawberry intake. Food Chem. 117, 181–188 (2009).

[b28] Tulipani S. *et al.* Antioxidants, phenolic compounds, and nutritional quality of different strawberry genotypes. J. Agric. Food Chem. 56, 696–704 (2008). 18211027 10.1021/jf0719959

[b29] Steeg P. S. Tumor metastasis: mechanistic insights and clinical challenges. Nat. Med. 12, 895–904 (2006). 16892035 10.1038/nm1469

[b30] Capocasa F., Scalzo J., Mezzetti B. & Battino M. Combining quality and antioxidant attributes in the strawberry: the role of genotype. Food Chem. 111, 872–878 (2008).

[b31] Tulipani S. *et al.* Influence of environmental and genetic factors on health-related compounds in strawberry. Food Chem. 124, 906–913 (2011).

[b32] Lopes-da-Silva F., de Pascual-Teresa S., Rivas-Gonzalo J. C. & Santos-Buelga C. Identification of anthocyanin pigments in strawberry (cv. Camarosa) by LC using DAD and ESI-MS detection. Eur. Food Res. Technol. 214, 248–253 (2002).

[b33] Yokoyama T. *et al.* Serum vitamin C concentration was inversely associated with subsequent 20-year incidence of stroke in a Japanese rural community. The Shibata study. Stroke 31(10), 2287–2294 (2000). 11022052 10.1161/01.str.31.10.2287

[b34] Steinmetz K. A. & Potter J. D. Vegetables, fruit, and cancer prevention: a review. J. Am. Diet. Assoc. 96(10), 1027–1039 (1996). 8841165 10.1016/S0002-8223(96)00273-8

[b35] Houston D. K. & Johnson M. A. Does vitamin C intake protect against lead toxicity? Nutr. Rev. 58, 73–75 (2000). 10812921 10.1111/j.1753-4887.2000.tb01842.x

[b36] Sapei L. & Hwa L. Study on the Kinetics of Vitamin C Degradation in Fresh Strawberry Juices. Procedia Chemistry 9, 62–68 (2014).

[b37] Giampieri F. *et al.* An anthocyanin-rich strawberry extract protects against oxidative stress damage and improves mitochondrial functionality in human dermal fibroblasts exposed to an oxidizing agent. Food Funct. 5(8), 1939–1948 (2014). 24956972 10.1039/c4fo00048j

[b38] Giampieri F. *et al.* Polyphenol-Rich Strawberry Extract Protects Human Dermal Fibroblasts against Hydrogen Peroxide Oxidative Damage and Improves Mitochondrial Functionality. Molecules 19, 7798–7816 (2014). 24962387 10.3390/molecules19067798 PMC6270910

[b39] Romandini S. *et al.* Effects of an acute strawberry (Fragaria × ananassa) consumption on the plasma antioxidant status of healthy subjects. J. Berry Res. 3, 169–179 (2013).

[b40] Alvarez-Suarez J. M. *et al.* One-month strawberry-rich anthocyanin supplementation ameliorates cardiovascular risk, oxidative stress markers and platelet activation in humans. J. Nutr. Biochem. 25(3), 289–294 (2014). 24406274 10.1016/j.jnutbio.2013.11.002

[b41] Marchini C. *et al.* Mesenchymal/stromal gene expression signature relates to basal-like breast cancers, identifies bone metastasis and predicts resistance to therapies. PLoS One 5(11), e14131 (2010). 21152434 10.1371/journal.pone.0014131 PMC2994727

[b42] Qin L. *et al.* NCOA1 directly targets M-CSF1 expression to promote breast cancer metastasis. Cancer Res. 74(13), 3477–3488 (2014). 24769444 10.1158/0008-5472.CAN-13-2639 PMC4083628

[b43] Zeng G. F., Cai S. X. & Wu G. J. Up-regulation of METCAM/MUC18 promotes motility, invasion, and tumorigenesis of human breast cancer cells. BMC Cancer 11, 113 (2011). 21450088 10.1186/1471-2407-11-113 PMC3079690

[b44] Yuan Z. Y. *et al.* Overexpression of ETV4 protein in triple-negative breast cancer is associated with a higher risk of distant metastasis. Onco. Targets Ther. 7, 1733–1742 (2014). 25328406 10.2147/OTT.S66692 PMC4196788

[b45] Loganathan J. *et al.* The mushroom Ganoderma lucidum suppresses breast-to-lung cancer metastasis through the inhibition of pro-invasive genes. Int. J. Oncol. 44(6), 2009–2015 (2014). 24718855 10.3892/ijo.2014.2375 PMC4735696

[b46] Zhao J. *et al.* TIP30/CC3 expression in breast carcinoma: relation to metastasis, clinicopathologic parameters, and P53 expression. Hum. Pathol. 38(2), 293–298 (2007). 17097132 10.1016/j.humpath.2006.08.005

[b47] Rose A. A. & Siegel P. M. Osteoactivin/HGFIN: is it a tumor suppressor or mediator of metastasis in breast cancer? Breast Cancer Res. 9(6), 403 (2007). 18086324 10.1186/bcr1791 PMC2246174

[b48] Tholen M. *et al.* Stress-resistant translation of Cathepsin L mRNA in breast cancer progression. J. Biol. Chem. 290(25), 15758–15769 (2015). 25957406 10.1074/jbc.M114.624353 PMC4505485

[b49] Okuyama Kishima M. *et al.* Immunohistochemical expression of CXCR4 on breast cancer and its clinical significance. Anal. Cell Pathol. (Amst) 2015, 891020 (2015). 26161302 10.1155/2015/891020 PMC4486754

[b50] Carter R. Z. *et al.* Tumour but not stromal expression of β3 integrin is essential, and is required early, for spontaneous dissemination of bone-metastatic breast cancer. J. Pathol. 235(5), 760–772 (2015). 25430721 10.1002/path.4490

[b51] Bodey B., Bodey B. Jr., Siegel S. E. & Kaiser H. E. Matrix metalloproteinases in neoplasm-induced extracellular remodeling in breast carcinomas. Anticancer Res. 21(3B), 2021–2028 (2001). 11497292

[b52] Re R. *et al.* Antioxidant activity applying an improved ABTS radical cation decolorization assay. Free Radic. Biol. Med. 26, 1231–1237 (1999). 10381194 10.1016/s0891-5849(98)00315-3

[b53] Pellegrini N., Re R., Yang M. & Rice-Evans C. A. Screening of dietary carotenoids and carotenoid-rich fruit extracts for antioxidant activities applying the 2,2‟-azobis(3-ethylenebenzothiazoline-6-sulfonic) acid radical cation decolorization assay. Method Enzymol. 299, 379–389 (1999).

[b54] Deighton N., Brennan R., Finn C. & Davies H. V. Antioxidant properties of domesticated and wild Rubus species. J. Sci. Food Agric. 80, 1307–1313 (2000).

[b55] Benzie I. F. F. & Strain J. J. Ferric reducing ability of plasma (FRAP) as a measure of antioxidant power: The FRAP assay. Anal. Biochem. 239, 70–76 (1996). 8660627 10.1006/abio.1996.0292

[b56] Slinkard K. & Singleton V. L. Total Phenol analysis: automation and comparision with manual methods. Am. J. Enol. Viticult. 28, 49–55 (1977).

[b57] Giusti M. M. & Wrolstad R. E. Characterization and measurement of anthocyanins by UV-visible spectroscopy in Current Protocols Food Analytical Chemistry, F1.2.1–F1.2.13 (John Wiley & Sons, Inc 2001).

[b58] Jia Z., Tang M. & Wu J. The determination of flavonoid contents in mulberry and their scavenging effects on superoxides radicals. Food Chem. 64, 555–559 (1998).

[b59] Dewanto V., Wu X., Adom K. K. & Liu R. H. Thermal processing enhances the nutritional values of tomatoes by increasing the total antioxidant activity. J. Agric. Food Chem. 50, 3010–3014 (2002). 11982434 10.1021/jf0115589

[b60] Helsper J. P. F. G. *et al.* Response of selected antioxidants and pigments in tissues of Rosa hybrida and Fuchsia hybrida to supplemental UV-A exposure. Physiol Plant. 117, 171–187 (2003).

[b61] Fanelli M. *et al.* Loss of pericentromeric DNA methylation pattern in human glioblastoma is associated with altered DNA methyltransferases expression and involves the stem cell compartment. Oncogene 27(3), 358–365 (2008). 17653095 10.1038/sj.onc.1210642

[b62] Puppo F. *et al.* Cell-line specific chromatin acetylation at the Sox10–Pax3 enhancer site modulates the RET proto-oncogene expression. FEBS Lett. 523(1–3), 123–127 (2002). 12123817 10.1016/s0014-5793(02)02957-5

[b63] Chou T. C. Theoretical basis, experimental design, and computerized simulation of synergism and antagonism in drug combination studies. Pharmacol. Rev. 58, 621–681 (2006). 16968952 10.1124/pr.58.3.10

[b64] Amatori S. *et al.* Decitabine, differently from DNMT1 silencing, exerts its antiproliferative activity through p21 upregulation in malignant pleural mesothelioma (MPM) cells. Lung Cancer 66(2), 184–190 (2009). 19233506 10.1016/j.lungcan.2009.01.015

[b65] Kalogris C. *et al.* Sanguinarine suppresses basal-like breast cancer growth through dihydrofolate reductase inhibition. Biochem. Pharmacol. 90(3), 226–234 (2014). 24875448 10.1016/j.bcp.2014.05.014

[b66] Amatori S. *et al.* Malten, a new synthetic molecule showing *in vitro* antiproliferative activity against tumour cells and induction of complex DNA structural alterations. Br. J. Cancer 103(2), 239–248 (2010). 20571494 10.1038/sj.bjc.6605745 PMC2906739

